# A Conceptual Classification of Resectability for Hepatocellular Carcinoma

**DOI:** 10.1007/s00268-022-06803-7

**Published:** 2022-10-26

**Authors:** Tomoaki Yoh, Takamichi Ishii, Takahiro Nishio, Yukinori Koyama, Satoshi Ogiso, Ken Fukumitsu, Yoichiro Uchida, Takashi Ito, Satoru Seo, Koichiro Hata, Etsuro Hatano

**Affiliations:** Department of Surgery, Graduate School of Medicine, Kyoto University, 54 Kawahara-Cho, Shogoin, Sakyo-Ku, Kyoto, 606-8507 Japan

## Abstract

**Backgrounds:**

In the era of multidisciplinary treatment strategy, resectability for hepatocellular carcinoma (HCC) should be defined. This study aimed to propose and validate a resectability classification of HCC.

**Methods:**

We proposed following the three groups; resectable-(R), borderline resectable-(BR), and unresectable (UR)-HCCs. Resectable two groups were sub-divided according to the value of indocyanine green clearance of remnant liver (ICG-Krem) and presence of macrovascular invasion (MVI); BR-HCC was defined as resectable HCCs with MVI and/or ICG-Krem≥0.03–<0.05, and R-HCC was the remaining. Consecutive patients with HCC who underwent liver resection (LR) and non-surgical treatment(s) (i.e., UR-HCC) between 2011 and 2017 were retrospectively analyzed to validate the proposed classification.

**Results:**

A total of 361 patients were enrolled in the study. Of these, R-, BR- and UR-HCC were found in 251, 46, and 64 patients, respectively. In patients with resected HCC, ICG-Krem≥0.05 was associated with decreased risk of clinically relevant posthepatectomy liver failure (*p*=0.013) and the presence of MVI was associated with worse overall survival (OS) (*p*<0.001). The 3–5-years OS rates according to the proposed classification were 80.3, and 68.3% versus 51.4, and 35.6%, in the R and BR groups, respectively (both *p*<0.001). Multivariate analysis showed BR-HCC was independently associated with poorer OS (*p*<0.001) after adjusting for known tumor prognostic factors. Meanwhile, BR-HCC was associated with benefit in terms of OS compared with UR-HCC (*p*<0.001).

**Conclusion:**

Our proposal of resectability for HCC allows for stratifying survival outcomes of HCC and may help to determine treatment strategy.

**Supplementary Information:**

The online version contains supplementary material available at 10.1007/s00268-022-06803-7.

## Introduction

Hepatocellular carcinoma (HCC) is the most common primary liver malignancy, showing a worldwide increasing incidence [1, 2]. Among the available treatment options, liver resection (LR) is one of the most common treatments which yields long-term survival for selected patients with HCC [3, 4]. Especially, aggressive surgical procedures such as vascular resection/reconstruction may allow for achieving R0 resection in advanced cases; however, long-term outcomes for these patients still remain unsatisfactory [5]. Therefore, further improvement of the treatment strategy would be required.

To refine the treatment strategy, we would like to propose a concept of “resectability” for HCC. The concept of resectability was initiated in patients with pancreatic cancer and might allow for developing a new treatment strategy [6]. Resectability classification consists of three categories; resectable (R), borderline resectable (BR), and unresectable (UR) diseases [7]. Of these, the definition of BR should be debatable and BR seems to comprise surgical/anatomical complexity as well as oncological disadvantages [6, 7]. In other words, BR tumors represent worse survival than R tumors and surgery may provide limited benefit for patients with BR tumors in current practice; meanwhile, BR tumors require more complicated surgical technique to achieve R0 resection than R tumors (i.e., risk of residual tumor).

**Fig. 1:**
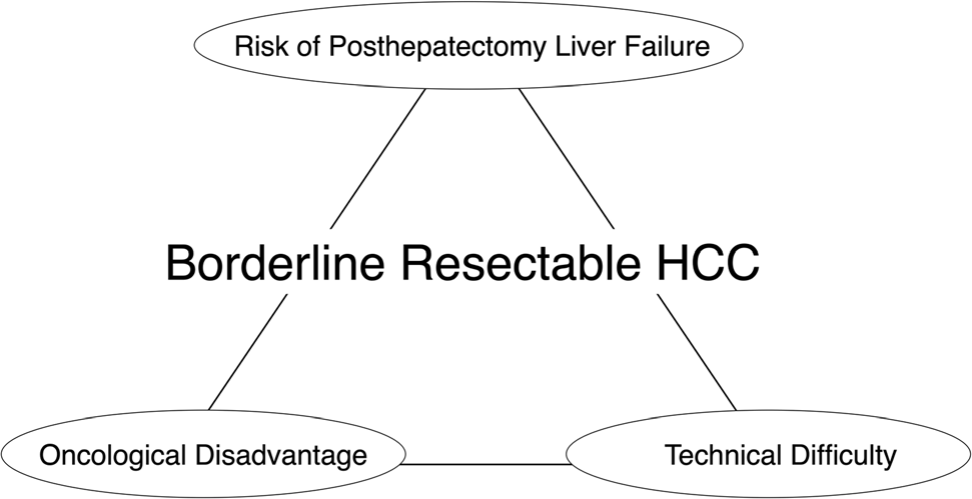
Fundamental concept of borderline resectable HCC. *Abbreviations: HCC* hepatocellular carcinoma

In the era of multidisciplinary treatment, the concept of resectability may have a potential to develop a new treatment strategy in patients with HCC; however, its application for HCC remains ill-defined. The main reason was that the balance between liver function and tumor progression should be taken into consideration for HCC. To date, some criteria combining liver and tumor factors (e.g., Barcelona Clinic Liver Cancer staging [1], or Cancer of the Liver Italian Program score [8]) have been adopted in the clinical practice; however, these criteria were not specified in patients undergoing LR. In the context of focusing on LR-specific population, we propose BR-HCC as a high-risk group of posthepatectomy liver failure (PHLF) and/or advanced disease (Fig. 1); indocyanine green clearance of remnant liver (ICG-Krem) and macrovascular invasion (MVI) were selected as determinants of BR-HCC based on our previous data, published series and practical guidelines [1, 2, 9–14]. The aim of the present study was to propose a classification of resectability for HCC combining these two factors and assess whether this classification could stratify the survival outcomes in patients with HCC.

## Methods

### Study design

The protocol of this retrospective observational study was approved by Kyoto University Graduate School and Faculty of Medicine, Ethics Committee (approval code: R3013). Written informed consent was obtained from all study participants.

### Proposal of resectability classification for HCC

**Fig. 2:**
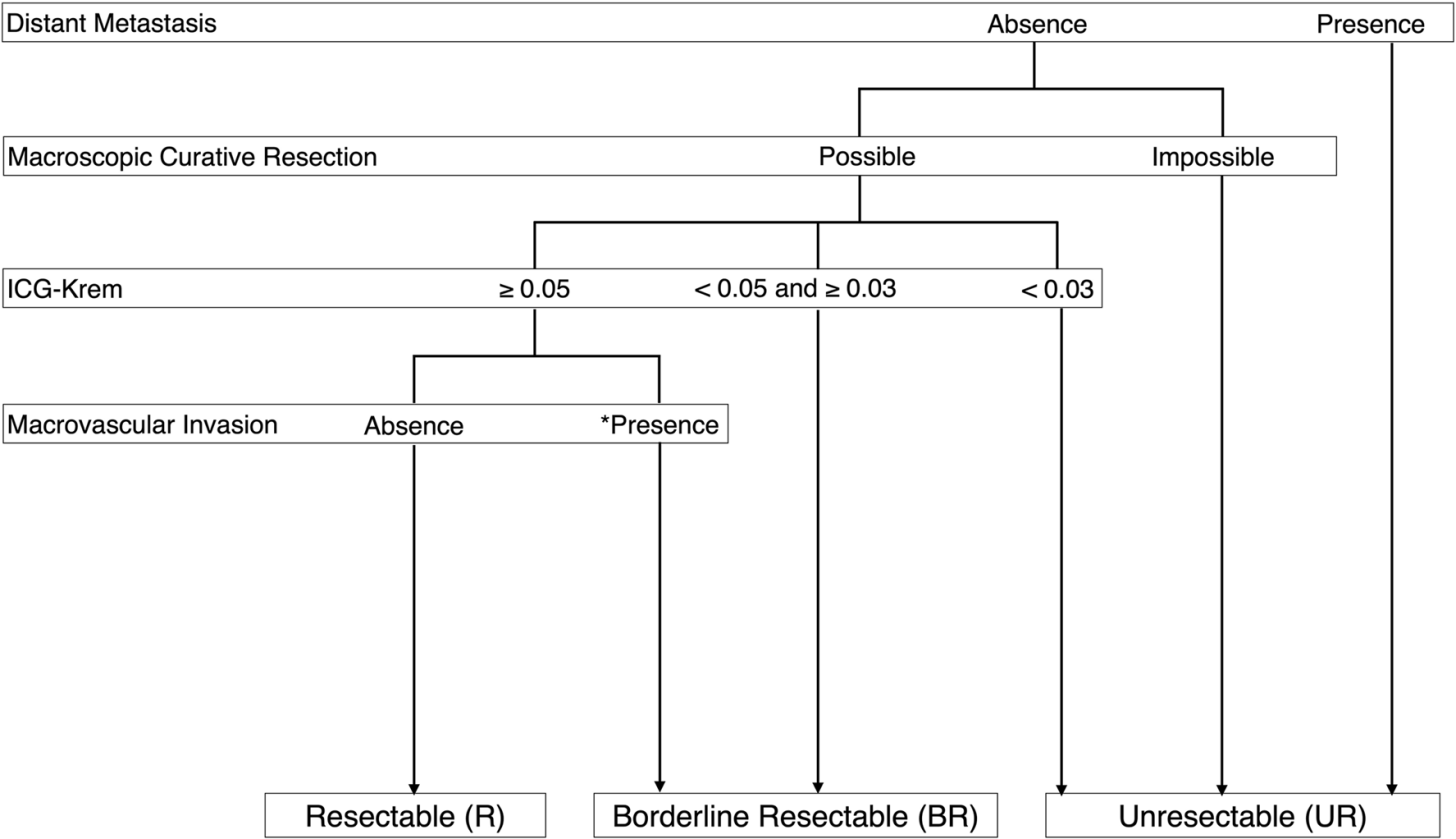
Proposed resectability classification of HCC. ^*^Macrovascular invasion was defined as involvement of Vp2-Vp4 and/or Vv2-Vv3 according to the Japanese staging system [17]. *Abbreviations: HCC* hepatocellular carcinoma

Our conceptual classification of resectability for HCC is shown in Fig. 2. UR-HCC was defined as the disease with distant metastasis or inability for macroscopic curative resection. Among the resectable disease, BR-HCC was defined as a high-risk group of clinically relevant PHLF (CR-PHLF) assessed by ICG-Krem and/or HCC with MVI (HCC-MVI); R-HCC was the remaining.

Risk assessment of PHLF should be prioritized, and a combination of the future liver remnant volume (FLRV) and the parameter of liver functional preserve may be its determinant [15]. ICG-Krem, which most hepatobiliary centers routinely measure in Japan, simultaneously balances these two aspects and therefore we selected it as an indicator of a high-risk group of CR-PHLF. ICG-Krem was calculated using the following formula with reference to a previous study [13]: ICG-Krem=preoperative ICG-K (ICG clearance)×FLRV / total liver volume (TLV). In our center, ICG-Krem<0.03 was defined as contraindication for LR (i.e., UR-HCC) [10]; ICG-Krem<0.05–≥0.03 was set as high-risk of CR-PHLF according to the previous studies (i.e., BR-HCC) [10, 13, 14].

We also selected MVI as an indicator of tumor progression based on the fact that MVI represents surgical/anatomical complexity as well as oncological disadvantages [1, 2, 9, 16]. MVI was defined as involvement of Vp2-Vp4 and/or Vv2-Vv3 according to the Japanese staging system [9, 17], where Vp2, Vp3, and Vp4 represent portal vein tumor thrombosis (PVTT) in the second-order branch, first-order branch, and main trunk/contralateral branch of the portal vein, respectively; where Vv2 and Vv3 represent hepatic vein tumor thrombosis (HVTT) in the first branch of the hepatic vein and inferior vena cava (IVC), respectively. From the technical aspects [18, 19] and the Hong Kong Liver Cancer classification [20], MVI was further classified into intra- (i.e., Vp2, Vp3, and Vv2) and extra-hepatic (i.e., Vp4 and Vv3) ones.

### Patients

We reviewed a prospectively maintained institutional database of consecutive patients who underwent LR (LR study population) and non-surgical treatment(s) for HCC at the Division of Hepatobiliary-Pancreatic Surgery and Transplantation, Department of Surgery, Kyoto University, between January 2011 and December 2017.

In the LR study population, the inclusion criteria were (1) patients with histologically diagnosed HCC. Exclusion criteria were (1) patients with apparent distant metastasis detected on computed tomography (CT), magnetic resonance imaging, ^18^F-fluorodeoxyglucose positron emission tomography or intraoperatively; (2) patients unavailable for prospective CT volumetry and/or ICG measurement; (3) patients who underwent repeated LR during the study period; (4) patients who underwent LR and concomitant ablative therapy because it was impossible for calculating actual ICG-Krem. Patients were divided into the two groups; R- and BR-HCC groups. Our database also included patients with HCC who were referred to our department to explore resectability, but eventually not indicated for LR; these patients were used as a control subject (i.e., UR-HCC group).

Clinicopathological data including sex, age, hepatitis virus markers, treatment-related variables, and survival data were retrieved from the database. Tumor characteristics including MVI were determined by the final clinicopathological findings. Routinely, ICG was administered intravenously at a dose of 0.5 mg/kg for assessing liver function within 2 weeks before LR. For the volume assessment, volumetric computed tomography was performed using a Virtual Place Lexus workstation (AZE, Tokyo, Japan) and SYNAPSE VINCENT (FUJITSU Tokyo) [10, 21]. Postoperative complications were defined according to the Clavien-Dindo classification [22]; complication was defined Clavien-Dindo≥grade II. CR-PHLF was defined as PHLF≥grade B according to the classification of the International Study Group of Liver Surgery (ISGLS) [23]. The follow-up protocol was previously reported [24].

### Surgical strategy of LR for HCC

The indications for LR included a CP grade of A or B, and ICG-Krem<0.03 is defined as a contraindication. Surgical procedures of LR were reported elsewhere [24, 25]. During the study period, postoperative hepatic arterial infusion therapy (HAIC) was administered for Vp3-Vp4 cases if appropriate [11]. In cases of advanced Vv3, preoperative HAIC rather than immediate resection was firstly considered [12]. Sorafenib, radiation therapy, and transcatheter arterial chemoembolization were added as supportive treatments for HAIC. Advanced Vv3 was defined as the following: (1) suspected extrahepatic metastasis; (2) the expected need for extracorporeal circulation, (e.g., right atrial tumor thrombus); (3) marginal liver function (i.e., ICG-Krem<0.05); and 4) multiple bilobar tumors. During the study period, postoperative sorafenib was administrated for patients with HVTT if appropriate [12].

### Statistical analysis

Categorical variables were analyzed with Chi-square test and Fisher’s exact test as appropriate. Continuous variables were analyzed using the Mann–Whitney *U* test. Overall survival (OS) was calculated from the date of operation (R- and BR-HCC) or the date of admission (UR-HCC) until death due to any cause, or the date of the last follow-up. Recurrence-free survival (RFS) was calculated from the date of the operation (R- and BR-HCC only) until the date of recurrence, or any cause of death. Survival curves were estimated using the Kaplan–Meier method, and a comparison was performed with the long-rank test. A multivariate analysis was performed using the Cox regression model using selected variables for variables with *p*<0.100 in univariate analysis. When collinearity was encountered, a choice was made based on the *p* value and clinical reasoning. All *p* values were two-sided and values less than 0.050 were considered statistically significant. Statistical analyses were performed using JMP Pro14.1 software (SAS Institute Inc., Cary, NC).

## Results

**Fig. 3:**
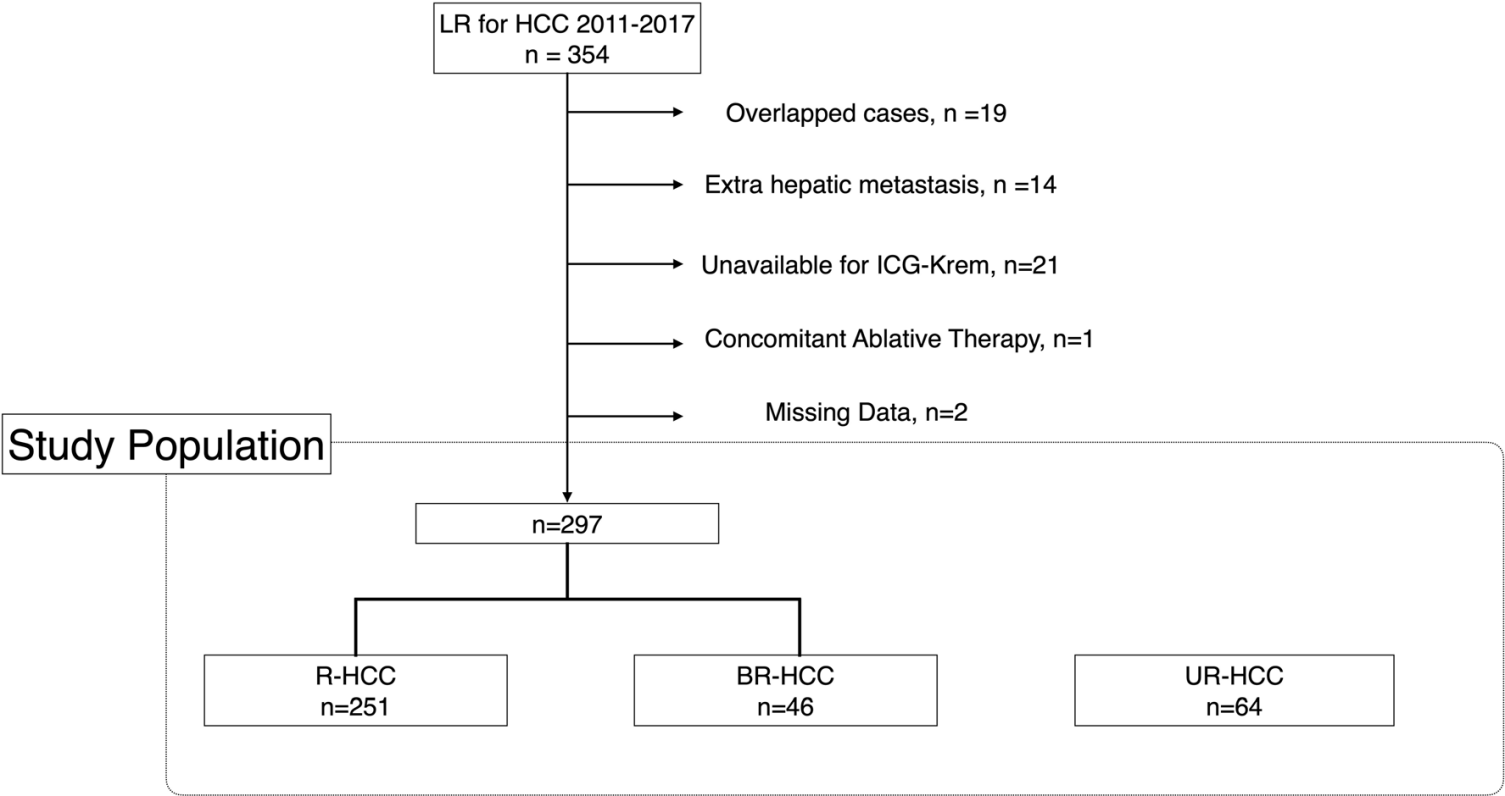
Flowchart of the study. *Abbreviations: LR* liver resection*, HCC* hepatocellular carcinoma*, ICG-Krem* indocyanine green clearance of remnant liver*, R-HCC* resectable HCC*, BR-HCC* borderline resectable HCC*, UR-HCC* unresectable HCC

Figure 3 shows a flow diagram of the present study. Between 2011 and 2017, 354 hepatectomies for HCC were performed. According to the study definition, a total of 297 patients were included in the LR study; of these, 251 and 46 patients were found in the R- and BR-HCC groups, respectively. Patient demographics in the LR study population are shown in Table 1. Meanwhile, 64 patients were identified as UR-HCC. Patient demographics in the UR-HCC group are shown in Supplementary Table 1.

**Table 1:** Patients demographics

Variables	Overall cohort *n*=297	R-HCC *n*=251	BR-HCC *n*=46	*P* value
*Preoperative findings*				
Age, median (range)	70 (20–88)	71 (20–88)	66 (40–82)	0.002*
Gender, male, *n* (%)	234 (78.8)	195 (77.7)	39 (84.8)	0.279
HBV (+), *n* (%)	59 (19.9)	45 (17.9)	14 (30.4)	0.051
HCV (+), *n* (%)	111 (37.4)	98 (39.0%)	13 (28.3)	0.165
Child-Pugh grade B, *n* (%)	18 (6.1)	12 (4.8)	6 (13.0)	0.043*^a^
Preoperative PVE	12 (4.0)	6 (2.40)	6 (13.0)	0.005*^a^
ICG-K, median (range)	0.139 (0.051–0.369	0.139 (0.051–0.369)	0.123 (0.052–0.217)	0.017*
FLRV/TLV (%), median (range)	77.5 (29.6–99.6)	81.7 (34.4–99.6)	59.2 (29.6–93.6)	<0.001*
ICG-Krem, median (range)	0.099 (0.036–0.345)	0.105 (0.050–0.345)	0.070 (0.036–0.134)	<0.001*
AFP levels (ng/ml), median (range)	12.5 (0.9–551,848)	10.0 (0.9–551,848)	267.0 (2.0–261,444)	<0.001*
*Surgery-related factors*				
Major liver resection≥3 segments^b^, *n* (%)	113 (38.1)	73 (29.1)	40 (87.0)	<0.001*
Operation time (min), median (range)	404 (93–975)	384 (93–961)	505 (234–975)	<0.001*
Blood loss (ml), median (range)	532 (0–9000)	430 (0–9000)	1518 (191–7610)	<0.001*
Complications, *n* (%)^c^	89 (30.0)	67 (26.7)	22 (47.8)	0.004*
CR-PHLF	31 (10.4)	21 (8.4)	10 (21.7)	0.015*^a^
Mortality<90 days^d^, *n* (%)	6 (2.0%)	5 (2.0%)	1 (2.2%)	1.000^a^
*Pathological findings*				
Vascular invasion	84 (28.3)	44 (17.5)	40 (87.0)	<0.001*
*Tumor number*				
solitary, *n* (%)	213 (71.3)	187 (74.5)	26 (56.5)	0.007*^a^
2–3 tumors, *n* (%)	63 (21.5)	51 (20.3)	12 (26.1)	
4 tumors, *n* (%)	21 (7.2)	13 (5.2)	8 (17.4)	
Maximum tumor diameter, median (range)	3.5 (0.7–20.0)	3.0 (0.7–20.0)	7.7 (1.6–17.0)	<0.001*
Poorly or un- differentiation, *n* (%)	88 (29.6)	58 (23.1)	30 (65.2)	<0.001*
R0 resection, *n* (%)	279 (93.9)	238 (94.8)	41 (89.1)	0.171^a^

*HBV* hepatitis B virus*, HCV* hepatitis C virus*, CP grade* Child-Pugh grade*,PVE* portal vein embolization*, ICG-K* indocyanine green clearance*, FLRV* future liver remnant volume*, TLV* total liver volume*, ICG-Krem* indocyanine green clearance of remnant liver*, AFP* alfa-feto protein*, CR-PHLF* clinically relevant posthepatectomy liver failure

^*^Significantly different (*p*<0.05)

^a^Fisher exact test

^b^Defined according to the 2000 Brisbane classification of the International Hepato-Pancreato-Biliary Association [26]

^c^≥ Grade II complications according to the  Clavien-Dindo classification [22]

^d^Including three patients with cancer-associated death

### Validity to use ICG-Krem and MVI for defining BR-HCC

#### ICG-Krem < 0.05 is a significant risk factor of CR-PHLF

**Fig. 4:**
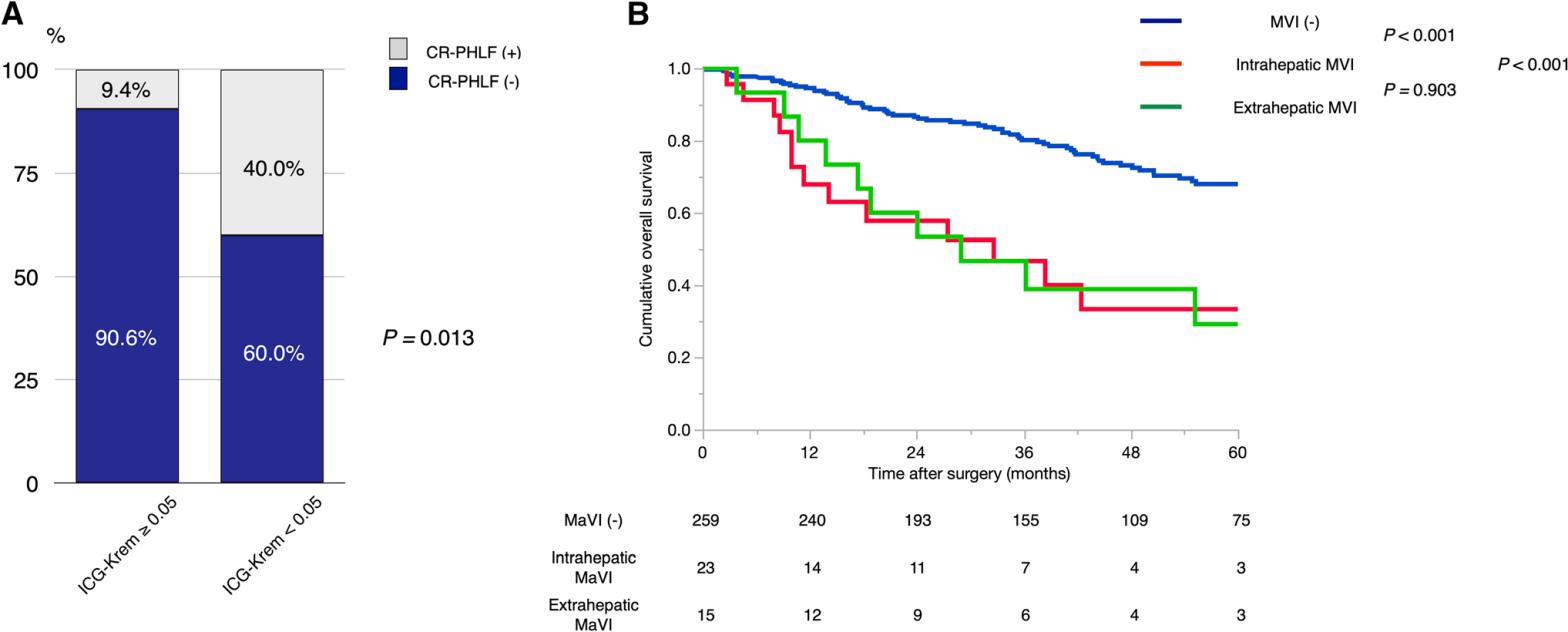
Validity of using ICG-Krem and MVI for resectability classification. *Abbreviations: ICG-Krem* indocyanine green clearance of remnant liver*, MVI* macrovascular invasion*, CR-PHLF* clinically relevant posthepatectomy liver failure

Median ICG-Krem was 0.099 (range, 0.036–0.345), and ICG-Krem<0.05 was found in 10 patients (3.3%). Of these, four patients (40%) preoperatively underwent portal vein embolization (PVE). No patients showed ICG-Krem<0.030 according to our strategy as aforementioned. Meanwhile, CR-PHLF was occurred in 31 patients (10.4%). ICG-Krem<0.05 was significantly associated with CR-PHLF (*p*=0.013, Fig. 4a).

#### Impact of MVI on survival outcomes in patients with HCC after LR

Next, we assess the survival outcomes in patients with MVI after LR. A total of 38 patients with HCC (12.8%) showed MVI; of these, 23 and 15 patients showed intrahepatic MVI (i.e., Vp2, Vp3, or Vv2) and extrahepatic MVI (i.e., Vp4 or Vv3), respectively. Meanwhile, 27 patients with HCC-MVI underwent some adjuvant therapie(s) (the main treatment; HAIC, *n*=22; sorafenib, *n*=5). Survival outcomes according to the grade of MVI are shown in Fig. 4b. Patients with MVI showed worse OS than those without (*p*<0.001); meanwhile, there was no significant difference with regards to OS between intrahepatic and extrahepatic MVIs **(***p*=0.903, Fig. 4b**).**

#### Survival outcomes according to the proposed classification

Clinicopathological characteristics between the R- and BR-HCC groups are also shown in Table 1. Compared with the R-HCC group, BR-HCC group showed younger age, worse liver function, worse perioperative outcomes and aggressive tumor characteristics.

**Fig. 5:**
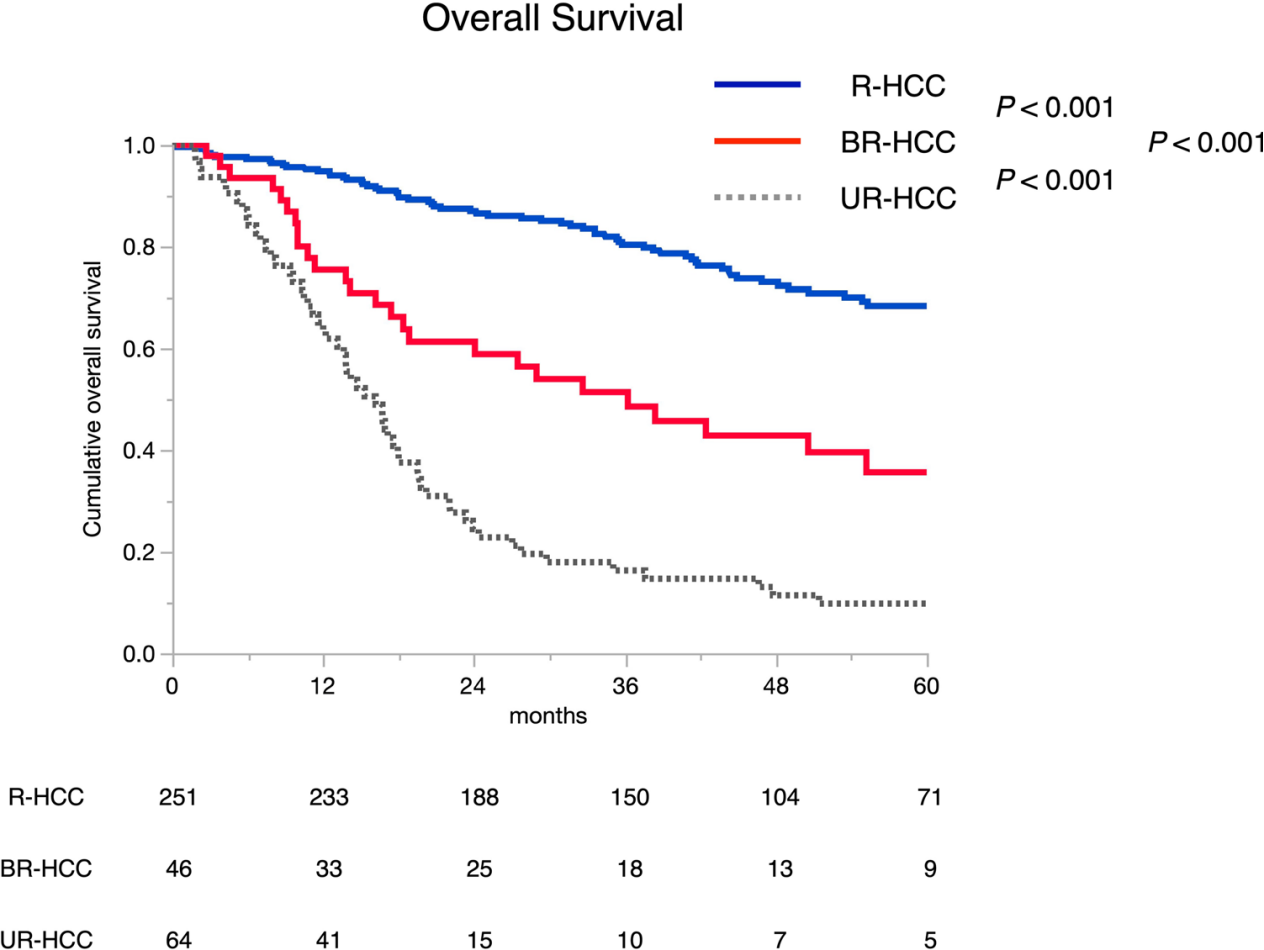
Overall survival stratified by the proposed resectability classification. *Abbreviations: HCC* hepatocellular carcinoma*, R-HCC* resectable HCC*, BR-HCC* borderline resectable HCC*, UR-HCC* unresectable HCC

The median OS time and the 1, 3, and 5-year OS rates of patients in the R-HCC group were significantly better compared with the BR-HCC group (not reached and 94.8, 80.3, and 68.3% vs. 36.2 months and 75.4, 51.4, and 35.6% for R- vs. BR-HCC groups, respectively, *p*<0.001; Fig. 5). In the UR-HCC group, the median OS time, and 1, 3, and 5-year OS rates were 16.2 months and 65.1%, 16.3%, and 9.8%, respectively. Better OS were found in both the R- and BR-HCC groups compared with the UR-HCC group (both *p*<0.001). The median RFS time and the 1, 3, and 5-year RF rates of patients in the R-HCC group were significantly better compared with the BR-HCC group (35.0 months, and 75.8%, 47.7% and 40.8% vs. 8.9 months, and 33.9, 23.5 and 20.1% for R- vs. BR-HCC groups, respectively, *p*<0.001, Supplemental Fig. 1).

To assess the prognostic independency of the proposed BR-HCC, multivariate analysis for OS was performed in the LR study population. Seven selected factors which might be preoperatively available were entered into the multivariate model. After adjusting for these factors, BR-HCC was independently associated with poorer OS (hazard ratio: 2.597, 95% confidence interval; 1.558–4.329, *p*<0.001**,** Table 2).

**Table 2:** Risk factors of poorer overall survival in patients who underwent liver resection for HCC

Variables		Univariate *p* value	Multivariate analysis		*p* value
			HR	95% CI	
Age	Every 1 year increase	0.428			–
Gender	Female	0.638			–
	Male				
Child-Pugh classification	Grade A	0.140			–
	Grade B				
Serum AFP levels	Every 10 ng/ml increase	0.384			–
	Solitary	Reference			
Tumor number	2–3 tumors	0.276			–
	≥4 tumors	0.315			
Maximum tumor diameter	Every 1 cm increase	0.004*	1.029	0.971–1.086	0.313
Resectability classification	R-HCC	<0.001*	Reference		<0.001*
	BR-HCC		2.597	1.558–4.329	

*HCC* hepatocellular carcinoma*, HR* hazard ratio*, CI* confidence interval*, R-HCC* resectable HCC*, BR-HCC* borderline resectable HCC

^*^Significantly different (*p*<0.05)

## Discussion

In the era of multidisciplinary approach or multiple choice of treatments for malignancies, refinement of surgical strategy for HCC would be required. In this context, we proposed a resectability classification of HCC which consisted of three categories; R-, BR-, and UR-HCCs. We defined BR-HCC, the most debatable subgroup, as a high-risk group of PHLF assessed by ICG-Krem and/or HCC-MVI. In this setting, a proposed resectability classification significantly stratified OS among the R-, BR-and UR-HCCs.

The concept of resectability was initiated in pancreatic cancer and has already been accepted worldwide [6, 7], yet, its application for HCC remains ill-defined. From a surgical perspective for HCC, liver function and tumor progression should be balanced. Of note, liver function should be prioritized the most to avoid postoperative mortality, and therefore, many guidelines prioritized assessment of liver function [1, 2]. According to our recent systematic review, preoperative FLRV and the status of portal hyper-pressure seem to determine the PHLF [15]. This study advocated the use of ICG-Krem. The reason was that measurement of ICG is a standard practice for determining surgical indication in liver surgery especially in Japan [27] and many investigators have reported that ICG-Krem<0.05 was a significant risk factor of PHLF [10, 13, 14].

Meanwhile, we focused on MVI as a tumor factor for resectability classification based on its anatomical/surgical complexity as well as poor prognosis [28, 29]. From the oncological aspect, the European Association for the Study of Liver disease guideline does not recommend LR for HCC-MVI; meanwhile, a Japanese national-wide study reported that LR for selected HCC-MVI (e.g., up to Vp3 in PVTT cases) might be associated with benefit compared with non-surgical therapy [28, 29]. This controversy prompted us to include MVI into the resectability classification of HCC. Although we investigated the prognostic impact of intrahepatic and extrahepatic MVIs based on the surgical difficulty or tumor aggressiveness [12, 18, 19], their prognostic value was comparable. Therefore, HCC-MVI would not be sub-divided. We have taken several efforts to improve the long-term outcomes of HCC-MVI; postoperative HAIC was administrated for Vp3-Vp4 cases to prevent early intrahepatic recurrence [11], and preoperative HAIC was administrated for advanced Vv3 to evaluate the biological aggressiveness because those who progress under chemotherapy may not benefit from LR [12]. These strategies were associated with benefit, suggesting LR and some additional therapy may have a potential to improve survival outcomes of these patients.

The definition of BR-HCC represents clinical relevance for developing a new treatment strategy or contribution to future prospective studies in the era of multidisciplinary treatment strategy. In this study, BR-HCC was identified as an only independent prognostic factor in patients with HCC who underwent LR, and therefore, our classification may have a potential for these perspectives. For one example, we propose neoadjuvant therapy for expecting downsizing the tumor or biological change especially for BR-HCC in line with pancreatic cancer [6, 7]. Downsizing the tumor may change surgical procedures, which may result in increasing the FLRV/TLV and decreasing the risk of PHLF. As demonstrated by a recent randomized control trial (RCT) [30], effective preoperative therapy can prolong survival in patients with HCC-MVI. Besides, it should be noted that neoadjuvant therapy has another aspect to identifying its good responders. Considering these aspects, neoadjuvant strategy is worthwhile to be considered to improve the outcomes of BR-HCC and future RCT may allow for determining the best neoadjuvant approach. For the other examples, macroscopically no-margin LR which prioritizes the liver function [31], or preoperative PVE to increase the FLRV [32] may be selected. Besides, non-surgical approach (e.g., systemic chemotherapy) may be considered according to the overall patients’ condition.

A recent retrospective study demonstrated a possible role of chemotherapy that might allow patients with UR-HCC to become eligible for LR (i.e., “conversion” LR) [33]. At a present, LR might provide benefit for selected patients in our practice (Fig. 5). Although UR-HCC represents a dismal prognosis, conversion LR may shed a light for these patients and clinicians should not miss the chance.

Several limitations of this study should be pointed out. The main limitation of this study was that this resectability classification was derived from a single institution’s experience. To assess the validity of this classification, we used a different cohort from previous ones [10–12]. To assess the further validity of our proposal, external studies would be required. Secondly, the true contribution of LR for BR-HCC on long-term survival remains unknown. This was also biased by a single institutional analysis. Lastly, the disadvantage of ICG clearance (e.g., flow-dependency or unavailability in some countries) may limit generalization of our proposal for overall the world. According to our recent systematic review [15], combination of the FLRV and the parameter of liver functional preserve determines the occurrence of PHLF; however, the most general parameter of liver function is not clarified. At present, we consider ICG-Krem is the most reproducible parameter for assessing risk of PHLF based on our experience and recent studies [10, 13–15]. When unavailable for ICG, alternative parameter(s) (e.g., platelet count, albumin levels, liver stiffness by elastography, etc. [15]) should be used and further studies would be required for this context. Despite these limitations, we believe this classification contributes to the development of a new treatment strategy and is worthwhile to be validated by other centers.

## Conclusions

Our proposal of resectability for HCC allows for stratifying survival outcomes of HCC. We hope the future prospective studies will be conducted using our resectability classification of HCC.

## Supplementary Information

Below is the link to the electronic supplementary material.

Supplementary file1 (DOCX 85 KB)
