# Correction: A Conceptual Classification of Resectability for Hepatocellular Carcinoma

**DOI:** 10.1007/s00268-022-06838-w

**Published:** 2022-11-23

**Authors:** Tomoaki Yoh, Takamichi Ishii, Takahiro Nishio, Yukinori Koyama, Satoshi Ogiso, Ken Fukumitsu, Yoichiro Uchida, Takashi Ito, Satoru Seo, Koichiro Hata, Etsuro Hatano

**Affiliations:** Department of Surgery, Graduate School of Medicine, Kyoto University, 54 Kawahara-Cho, Shogoin, Sakyo-Ku, Kyoto, 606-8507 Japan

## Correction to: World J Surg https://doi.org/10.1007/s00268-022-06803-7

The article "A Conceptual Classification of Resectability for Hepatocellular Carcinoma", written by Tomoaki Yoh et al., was originally published electronically on the publisher’s internet portal on October 26, 2022, without open access. With the author(s)’ decision to opt for Open Choice the copyright of the article changed on November 9, 2022, to © The Authors 2022 and the article is forthwith distributed under a Creative Commons Attribution 4.0 International License.

