# Supplementary material for: A Conceptual Classification of Resectability for Hepatocellular Carcinoma
Source: World J Surg. 2022 Oct 26;47(3):740–8. doi: 10.1007/s00268-022-06803-7 (PMC9895025; doi:10.1007/s00268-022-06803-7)

SUPPLEMENTAL FILE

A Conceptual Classification of Resectability For Hepatocellular Carcinoma

Tomoaki YOH, Takamichi ISHII, Takahiro NISHIO, Yukinori KOYAMA, Satoshi OGISO, Ken FUKUMITSU, Yoichiro UCHIDA, Takashi ITO, Satoru SEO, Koichiro HATA, Etsuro HATANO

**AFFILIATIONS**

[1] Department of Surgery, Graduate School of Medicine, Kyoto University, Kyoto, Japan

**Corresponding author:**

Etsuro HATANO, MD, PhD

Department of Surgery, Graduate School of Medicine, Kyoto University

54 Kawahara-cho, Shogoin, Sakyo-ku, Kyoto 606-8507, Japan

Tel: +81-75-751-3651

Fax: +81-75-751-3106

E-mail: etsu@kuhp.kyoto-u.ac.jp

| **SUPPLEMENTARY TABLE 1**  Demographics in patients with UR-HCC | |
| --- | --- |
| Variables | n = 64 |
| Age, median (range) | 70 (37-85) |
| Gender, male, n (%) | 51 (79.7) |
| HBV (+), n (%) | 12 (18.8) |
| HCV (+), n (%) | 30 (46.9) |
| CP-grade A, n (%) | 22 (60.9) |
| CP-grade B, n (%) | 22 (4.7) |
| CP-grade C, n (%) | 3 (33.4) |
| AFP levels (ng/ml), median (range) | 54 (1.4-1111390) |
| Multiple Bilobar tumors | 15 (23.4) |
| MVI | 19 (36.5) |
| Intrahepatic MVI | 5 (9.6) |
| Extrahepatic MVI | 14 (26.9) |
| Extrahepatic metastasis | 13 (20.3) |
| Main reason for unresectable |  |
| Distant extrahepatic metastasis | 13 (20.3) |
| Extrahepatic MVI | 11 (17.2) |
| Multiple bilobar tumor | 16 (25.0) |
| Insufficient liver function | 17 (26.6) |
| General condition | 7 (10.9) |
| Main treatment |  |
| HAIC | 15 (23.4) |
| TACE | 29 (45.3) |
| Systemic chemotherapy | 3 (4.7) |
| Ablative therapy | 2 (3.1) |
| Radiotherapy | 2 (3.1) |
| Palliative care / others | 13 (20.3) |
| *Abbreviations: UR-HCC, unresectable hepatocellular carcinoma; HBV, hepatitis B virus; HCV, hepatitis C virus; CP-grade, Child Pugh grade; AFP, alfa-feto protein; MVI, macrovascular invasion; HAIC, hepatic arterial infusion chemotherapy; TACE, transarterial chemoembolization* | |

**SUPPLEMENTARY FUGIRE 1**

Recurrence-free survival between R- and BR- HCCs

*Abbreviations, HCC, hepatocellular carcinoma; R-HCC, resectable HCC; BR-HCC, borderline resectable HCC*


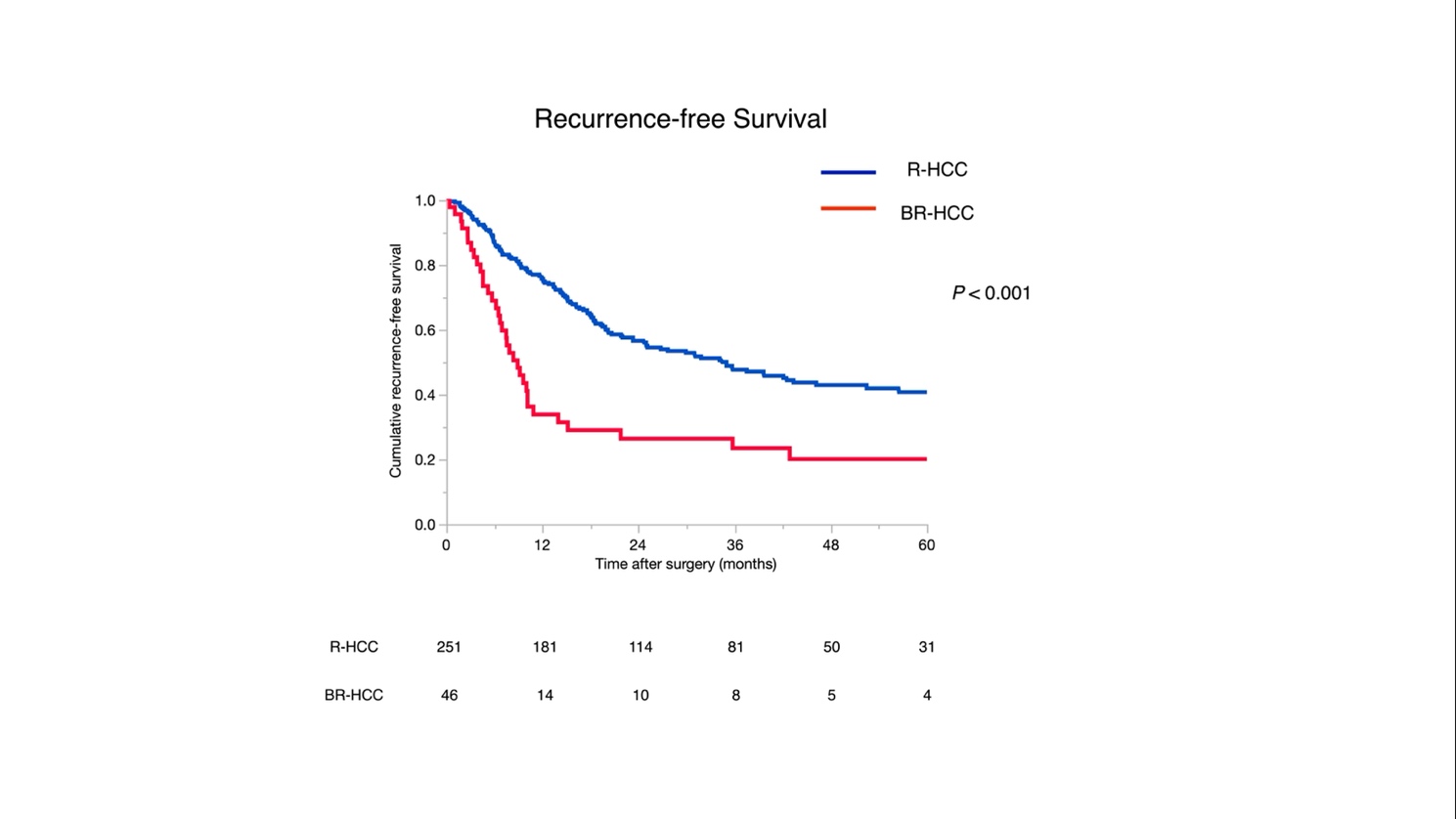

Supplement: Supplementary file 1 — Supplementary file1 (DOCX 85 KB) [file 268_2022_6803_MOESM1_ESM.docx]
